# Cryptochrome-dependent magnetic field effect on seizure response in *Drosophila* larvae

**DOI:** 10.1038/srep05799

**Published:** 2014-07-23

**Authors:** Richard Marley, Carlo N. G. Giachello, Nigel S. Scrutton, Richard A. Baines, Alex R. Jones

**Affiliations:** 1 Faculty of Life Sciences, The University of Manchester, Oxford Road, Manchester, M13 9PT, UK; 2 Manchester Institute of Biotechnology and Faculty of Life Sciences, The University of Manchester, 131 Princess Street, Manchester, M1 7DN; 3 Photon Science Institute and School of Chemistry, The University of Manchester, Oxford Road, Manchester, M13 9PL, UK; 4 These authors contributed equally to this work.

## Abstract

The mechanisms that facilitate animal magnetoreception have both fascinated and confounded scientists for decades, and its precise biophysical origin remains unclear. Among the proposed primary magnetic sensors is the flavoprotein, cryptochrome, which is thought to provide geomagnetic information *via* a quantum effect in a light-initiated radical pair reaction. Despite recent advances in the radical pair model of magnetoreception from theoretical, molecular and animal behaviour studies, very little is known of a possible signal transduction mechanism. We report a substantial effect of magnetic field exposure on seizure response in *Drosophila* larvae. The effect is dependent on cryptochrome, the presence and wavelength of light and is blocked by prior ingestion of typical antiepileptic drugs. These data are consistent with a magnetically-sensitive, photochemical radical pair reaction in cryptochrome that alters levels of neuronal excitation, and represent a vital step forward in our understanding of the signal transduction mechanism involved in animal magnetoreception.

Many animals sense the Earth's magnetic field. Of the proposed biophysical mechanisms, the best described are a magnetite-based system^1^,^2^ and chemical magnetoreception based on a photoinitiated radical pair reaction^3^,^4^. Both have credible experimental and theoretical foundations, and may not be mutually exclusive. Much of the behavioural work in this area has been conducted using complex animals that migrate (*e.g.* species of bird, turtle and lobster)^5^. However, simpler animals that don't migrate, including the fruit fly *Drosophila melanogaster*^6^,^7^,^8^,^9^,^10^, also possess a magnetic sense. This significantly broadens the type of biophysical, neurobiological and genetic investigation available to establish primary receptor mechanism and signal transduction. The magnetic sense of *Drosophila* is dependent on the presence of the flavin adenine dinucleotide (FAD)-containing, circadian clock photoreceptor protein, cryptochrome (*Dm*CRY)^7^, and the presence and wavelength of light to which the flies are exposed^6^,^7^,^8^,^9^. CRY are closely related to the light-dependent DNA repair enzymes, the photolyases. A second, UV-harvesting pterin chromophore is also present in members of the CRY/photolyase family, but the residues involved in binding differ significantly in *Dm*CRY such that pterin-binding is thought unlikely^11^,^12^,^13^. CRY-dependent magnetoreception is currently proposed to be a result of light-initiated electron transfer chemistry in the protein, which is magnetically-sensitive by virtue of the radical pair mechanism^3^,^4^. Spin correlated radical pairs can undergo coherent mixing between singlet and triplet spin states, which have different reactive fates, and this mixing process can be modulated by magnetic fields^14^,^15^. The exact identity of the magnetically-sensitive radical pair in CRY is currently unknown. Presumably the influence of the magnetic field in some way affects the concentration of a CRY signalling state that, in turn, results in a neurophysiological response. However, there exists very little evidence of the signal transduction mechanism that might link magnetically-sensitive chemistry in CRY to an organism response.

Fogle *et al.* have shown that expression of *Dm*CRY in central neurons in *Drosophila* is sufficient to bestow photosensitivity to those neurons, such that illumination with blue light (450–490 nm) increases action potential firing^16^. Thus, we hypothesise that a light-induced change to neuron activity levels, mediated by *Dm*CRY, might be modified by external magnetic fields. To date no physical mechanism in a primary magnetoreceptor (CRY or magnetite) has been demonstrated to unequivocally produce a magnetically-induced response in neuronal activity^17^,^18^. As part of a study investigating the importance of patterned activity for the development of robust neural circuitry in the developing *Drosophila* embryonic CNS, we noted that exposing embryos to pulsed blue light (~470 nm) resulted in a heightened seizure-phenotype when tested post-embryonically at the third instar larval stage. Such a phenotype has been associated with, and is an indicator of, increased synaptic excitation in the locomotor circuitry^19^,^20^. We show in this study, that the effect of blue light pulses during embryogenesis is significantly potentiated by the presence of a magnetic field. The effect of both light and applied magnetic field is blocked by prior ingestion of typical antiepileptic drugs, indicative of a change to neuronal activity level. Moreover, the effect of both light and light + magnetic field requires the presence of *Dm*CRY. Thus, we conclude that an applied magnetic field alters the ability of light-activated *Dm*CRY to influence levels of synaptic excitation in the *Drosophila* CNS.

## Results

**Figure 1:**
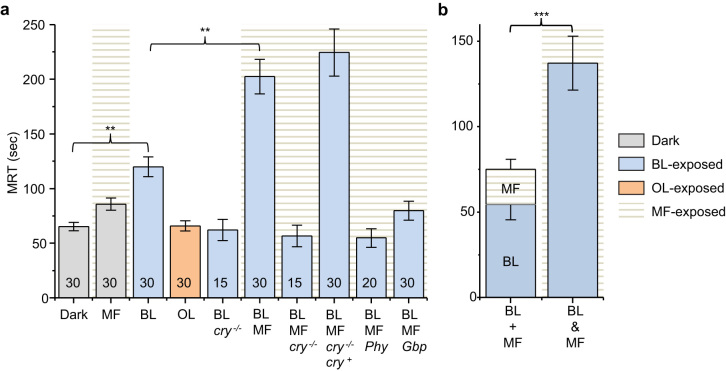
Seizure duration, measured as mean recovery time (MRT), of *Drosophila* third instar larvae from electric shock. (a) Third instar larvae developed from embryos exposed to various conditions between 11–19 h after egg laying at 25°C. Colours of bars represent the wavelength of visible light that embryos were exposed to and the presence of a MF is indicated by background horizontal lines. Dark control (Dark); dark + static 100 mT magnetic field (MF); pulsed 470 nm blue light (BL); pulsed 590 nm orange light (OL); pulsed 470 nm + *cry^03^* null (BL/*cry^−/−^*); pulsed 470 nm + static 100 mT magnetic field (BL/MF); pulsed 470 nm + static 100 mT + *cry^03^* null (BL/MF/*cry^−/−^*); pulsed 470 nm + static 100 mT + *cry^01/03^* null, rescued with expression of *Dm*CRY (BL/MF/*cry^−/−^*/*cry^+^*); pulsed 470 nm, + static 100 mT + anti-epileptic drug, phenytoin (BL/MF/*Phy*); pulsed 470 nm, + static 100 mT + anti-epileptic drug, gabapentin (BL/MF/*Gbp*). All values shown are means ± sem and n is shown in each bar. ** P ≤ 0.01. (b) The combined effect of BL and MF (BL&MF) is significantly larger than the additive effect of BL alone added to MF alone (BL + MF). Values shown are adjusted MRT values, derived by subtracting values obtained in dark controls. *** P ≤ 0.001.

To identify a magnetic field effect (MFE) on the CNS of *Drosophila*, we employed an established assay designed to probe how manipulation of neuronal activity during embryogenesis in *Drosophila* affects the probability of seizure in subsequent third instar larvae (~3 days later)^19^,^20^. Seizure duration is measured as the mean recovery time (MRT) of *Drosophila* larvae from a DC electric shock across the anterior-dorsal surface (approximating the position of the underlying CNS). Single gene mutations of the bang-sensitive (*i.e.* seizure-sensitive) grouping of *Drosophila* show a significantly extended MRT compared to wildtype. Electrophysiological analysis shows that this effect observed in larvae is associated with increased levels of synaptic excitation in the CNS of these mutants during embryogenesis^19^. Exposing wildtype embryos to pulsed blue light (470 nm, 100 ms on/900 ms off) during 11–19 h of embryogenesis (when the locomotor neural circuits form)^21^ results in subsequent larvae that show significantly increased seizure duration compared to control embryos that developed in constant darkness (using a 30 V/3 s electroshock, Figure 1a). We found this effect of light to be *Dm*CRY-dependent; it is neither observed in a *cry^03^* loss-of-function mutation (*cry^−/−^*), nor is it produced when using pulsed orange light of 590 nm peak wavelength (Figure 1a). It is known that light-activated *Dm*CRY results in increased action potential firing in *Drosophila* arousal neurons^16^, which we hypothesise is sufficient to destabilise the development of the CNS leaving it prone to seizure^19^.

Significantly, repeating these experiments in the presence of a 100 mT magnetic field from a pair of NeFeB permanent magnets during the same period of embryogenesis substantially increased the effect of blue light on seizure severity in larvae compared to light-pulses alone (Figure 1a). We reproduced this MFE when a different researcher conducted equivalent experiments using a different population of flies, a different blue LED (from the same manufacturer) and a different electric shock stimulator (10 V/1.5 s). Qualitatively similar relative mean recovery times were recorded: control, 35.0 ± 7.2 s; blue light, 76.1 ± 13.3 s (*P* = 0.02 *vs.* control); blue light + magnetic field, 139.0 ± 15.5 s (*P* = 0.003 *vs.* control and blue light). The MFE on seizure duration was shown also to be *Dm*CRY-dependent: being abolished in a *cry^03^* null (*cry^−/−^*) background and rescued by transgenic expression of UAS-*cry* in a *cry* null (BL/MF/*cry^−/−^*/*cry^+^*, Figure 1a). Prolongation of seizure duration was also prevented by prior ingestion of typical antiepileptic drugs (*e.g.* phenytoin and gabapentin), consistent with an effect on neuronal activity (Figure 1a).

Although magnetic field exposure in the absence of light results in a marginally longer MRT than for the dark controls, the difference is not statistically significant (*P* > 0.99). Moreover, the MRT after exposure to a combination of blue light and magnetic field (137.3 ± 15.7 sec) is significantly longer than the MRT after exposure to blue light alone added to the MRT after exposure to magnetic fields alone (75.1 ± 9.1 sec, Figure 1b). The MFE is therefore dependent on light and is not simply an additive effect. The effect of antiepileptic drugs (phenytoin and gabapentin) is a strong indication that the increased seizure after exposure to blue light and magnetic field is related to increased synaptic excitation in the CNS. This result therefore represents an important initial step in unravelling the neuronal circuitry involved in CRY-dependent magnetoreception in *Drosophila*.

## Discussion

We present a significant MFE on seizure duration in *Drosophila* larvae. These data were acquired using an established proxy measurement for perturbations to neuronal activity. A change in neuronal activity that results from the response of any primary magnetosensor is considered necessary to produce an organism response^17^,^18^. The effect we observe requires light and is *Dm*CRY-dependent, the spin dynamics of which are potentially magnetically-sensitive *via* a photochemical radical pair mechanism^3^,^4^. Indeed, both low (<5 mT) and moderate (5–30 mT) magnetic fields have been reported to produce changes in quantum yield of flavin semiquinone radicals in photoreceptor CRY from *Arabidopsis thaliana* (*At*CRY1)^22^.

By analogy, magnetically sensitive radical pair reaction dynamics in *Dm*CRY may influence the concentration of FAD^**·**−^.There is evidence to suggest that this oxidation state of the flavin activates the protein in its role as circadian photoreceptor^23^. Conformational changes in the C-terminal tail of *Dm*CRY, which is well placed to respond to the flavin oxidation state, were observed to be kinetically coupled to the single electron reduction (by light or chemically) of oxidised FAD to FAD^**·**−^. This conformational change appears to allow interaction with its partner protein, Timeless (TIM), which ultimately leads to degradation of TIM and resetting of the clock. Interestingly, *Dm*CRY was found to revert back to its dark state conformation with the same kinetics as flavin reoxidation^23^. Consistent with this picture is the observation that clock neurons overexpressing *Dm*CRY result in *Drosophila* with free-running circadian periods that show an enhanced response to a 300 µT applied MF^8^. However, the conserved triad of tryptophans thought to act as an electron transfer chain to the flavin to generate the photoinitiated radical pair are not necessary for *Dm*CRY-dependent magnetic orientation of adult flies^9^. Moreover, light-induced conformational changes have also been observed in *Dm*CRY where the flavin was reduced chemically to FAD^**·**−^ prior to illumination^24^, and in variants containing tryp-triad mutations^25^. The authors of these studies argue that photoexcitation alone (of FAD^**·**−^ or even oxidised FAD), without any subsequent electron transfer chemistry, might be sufficient to trigger activation of *Dm*CRY^24^,^25^,^26^.

Alternatively, MFs might influence radical pair photochemistry in *Drosophila* *via* a mechanism that is *independent* of the CRY-TIM interaction that initiates signal transduction in the circadian clock. Light-activated *Dm*CRY is also known to result in an increased firing rate of arousal neurons^16^. This pathway is a consequence of light initiated redox chemistry in *Dm*CRY (which is likely to proceed *via* radical pair intermediates) that modulates potassium channels and results in membrane depolarisation. The fact that the MFE we observe is negated by prior ingestion of anti-epileptic drugs indicates the magnetically-sensitive activity of *Dm*CRY is similarly impacting neuronal firing activity. However, whether our observations, or *Drosophila* magnetoreception in general, is dependent or independent of the flies' circadian clock is yet to be determined.

Exposing the embryos to a 100 mT magnetic field in the first instance has a range of benefits over the µT field exposures immediately relevant to animal magnetoreception. First, the radical pair mechanism predicts that fields of this magnitude will saturate the Zeeman effect of typical organic radical pairs^14^. This is likely to produce a magnetically-induced change in spin selective product yield and reaction kinetics that is larger than those expected from µT fields, and therefore might produce a larger physiological and organism response. Second, potential variations in background field are much less significant when using mT exposure conditions compared to µT conditions. Finally, the use of permanent magnets removes the confounding variables of vibration and heating that are possible when using the electromagnets necessary for µT exposure. These factors may have been significant in the history of conflicting reports in the context of biological MFEs from exposure to µT fields, which includes examples concerning the radical pair/CRY model of magnetoreception^27^,^28^. Moderate (mT) field exposure was therefore chosen as a rational and reliable starting point, which has provided us with greater confidence in the observed effect before µT exposure experiments are conducted.

Our results represent an important initial step in elucidating the signal transduction mechanism between the response of the putative primary magnetoreceptor, cryptochrome, and a behavioural response in a genetically tractable organism. This study paves the way for assessing the influence of the amplitude and orientation of Earth strength magnetic fields (~µT) on seizure duration in *Drosophila* larvae. Using similar methods to those employed by Fogle *et al.*^16^, we can also confirm whether the MFE observed here is mediated through light-dependent redox chemistry in *Dm*CRY that is known to increase action potential firing in central brain neurones. This combined approach will provide a platform from which to detail the underlying electrophysiology of *Drosophila* magnetoreception.

## Methods

Flies were maintained on standard corn meal medium at 25°C. Embryos were collected by allowing females to lay on grape-agar (Dutscher, Essex, UK) plates supplemented with a small amount of live yeast paste at 25°C. Flies used were Canton-S wildtype and *cry^03^* homozygotes^29^. For rescue of *Dm*CRY expression, the following stocks were crossed: ElaV^C144^-GAL4;;*cry^03^* females crossed to UAS-Dm*cry*;*cry^01^* males.

### Magnetic field and light exposure during embryogenesis

**Figure 2:**
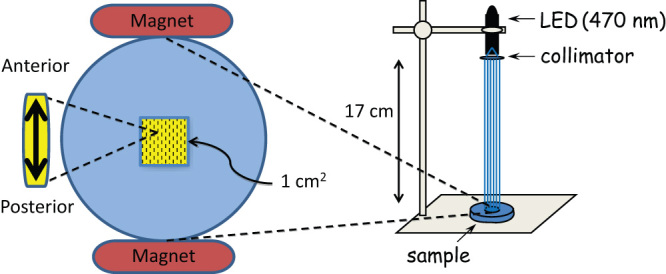
Schematic diagram of the light and magnetic field exposure apparatus used during embryogenesis. Embryos were aligned in a central region (1 cm^2^) on a grape-agar plate in rows of 10 such that all had the same anterior-posterior orientation, which was aligned parallel to the magnet separation axis. The plate was placed in a humidified atmosphere inside a 25°C incubator and exposed to collimated light from an overhead LED (*e.g.* 470 nm).

Embryos (~100, 1–3 h after egg laying) were aligned in a central region (1 cm^2^) on a grape-agar plate in rows of 10 such that all had the same anterior-posterior orientation, which was aligned, where applicable, parallel to the magnet separation axis (Figure 2). The magnetic field within the 1 cm^2^ region containing the embryos was measured to be 100 ± 5 mT. The plate was placed in a humidified atmosphere inside a 25°C incubator and, where applicable, exposed to collimated light from an overhead LED (Cairn Research Ltd, UK, Figure 2). LEDs were used with peak emission at 470 nm (bandwidth 25 nm, irradiance 466 ± 14 nW cm^−2^) or 590 nm (bandwidth 18 nm, 1094 ± 18 nW cm^−2^). Embryos were exposed to light for 100 ms every second between 11–19 h after egg laying, but exposed to the magnetic field throughout embryogenesis. After hatching, larvae were transferred to vials and maintained in complete darkness and in the absence of any applied magnetic field until ~3 days later when wall climbing third instar larvae were tested for seizure-like behaviour.

### Electroshock

Prior to stimulation, third instar larvae were washed to remove food residue and gently dried using paper tissue. Larvae were then allowed to recover on a plastic dish until normal crawling behaviour resumed. A stimulator, comprising two tungsten wires (0.1 mm diameter, ~1–2 mm apart) was placed across the anterior-dorsal surface, over the approximate position of the CNS. A DC pulse, generated by either a Grass S88 stimulator (30 V/3 s, Grass instruments, RI, USA) or constant current stimulator (10 V/1.5 s, DS2A, Digitimer, UK), was applied. The animal responded by tonically contracting and ceasing normal, motile behaviour. Time to resumption of normal motile behaviour was recorded (see [19] for more details). Results were analysed for significance using a one-way ANOVA with a Bonferroni *post-hoc* test.

### Drug-feeding

Mated adult females were fed with phenytoin (0.4 mg per ml) or gabapentin (0.1 mg per ml) for 2 days by adding flies to food vials containing the drug. Drugs (Sigma, UK) were prepared in DMSO, which has no effect on MRT^19^. After this period, flies were transferred to laying pots and embryos collected. After magnetic field and/or light exposure, newly hatched larvae were placed into drug-free vials and third instars tested at the wall-climbing stage as described above.

## Author Contributions

A.R.J. wrote the paper with N.S.S. and R.A.B. A.R.J. conceived and designed the method of magnetic field exposure during embryogenesis and R.A.B. and R.M. conceived and designed the light exposure conditions and electric shock assay. All experimental work was conducted by R.M and C.N.G.G. Data were analysed by R.M., C.N.G.G. and R.A.B. and results analysed by A.R.J., R.A.B. and N.S.S.
